# Crystal structure of pectocin M1 reveals diverse conformations and interactions during its initial step via the ferredoxin uptake system

**DOI:** 10.1002/2211-5463.13874

**Published:** 2024-08-09

**Authors:** Nawee Jantarit, Hideaki Tanaka, Yuxi Lin, Young‐Ho Lee, Genji Kurisu

**Affiliations:** ^1^ Protein Crystallography Laboratory, Institute for Protein Research Osaka University Suita Japan; ^2^ Department of Macromolecular Sciences, Graduate School of Science Osaka University Toyonaka Japan; ^3^ Biopharmaceutical Research Center Korea Basic Science Institute Ochang South Korea; ^4^ Bio‐Analytical Science University of Science and Technology Daejeon South Korea; ^5^ Graduate School of Analytical Science and Technology Chungnam National University Daejeon South Korea; ^6^ Department of Systems Biotechnology Chung‐Ang University Gyeonggi South Korea; ^7^ Frontier Research Institute for Interdisciplinary Sciences Tohoku University Sendai Japan; ^8^ Institute for Open and Transdisciplinary Research Initiatives (OTRI) Osaka University Suita Japan; ^9^ Institute of Science Suranaree University of Technology Nakohn Ratchasima Thailand

**Keywords:** bacteriocin, ferredoxin, lipid‐II‐degrading enzyme, *Pectobacterium*, siderophore, X‐ray crystallography

## Abstract

Pectocin M1 (PM1), the bacteriocin from phytopathogenic *Pectobacterium carotovorum* which causes soft rot disease, has a unique ferredoxin domain that allows it to use FusA of the plant ferredoxin uptake system. To probe the structure‐based mechanism of PM1 uptake, we determined the X‐ray structure of full‐length PM1, containing an N‐terminal ferredoxin and C‐terminal catalytic domain connected by helical linker, at 2.04 Å resolution. Based on published FusA structure and NMR data for PM1 ferredoxin domain titrated with FusA, we modeled docking of the ferredoxin domain with FusA. Combining the docking models with the X‐ray structures of PM1 and FusA enables us to propose the mechanism by which PM1 undergoes dynamic domain rearrangement to translocate across the target cell outer membrane.

## Graphical abstract

Pectocin M1 (PM1) contains ferredoxin‐like and colicin M‐like domains, enabling its transport via the ferredoxin uptake system (Fus) and facilitating peptidoglycan digestion against target cells, respectively. Based on the crystal structure of PM1 which allows domain rearrangement and diverse binding modes to the FusA receptor during the initial uptake, we propose a pectocin M (PM) uptake mechanism via Fus system.

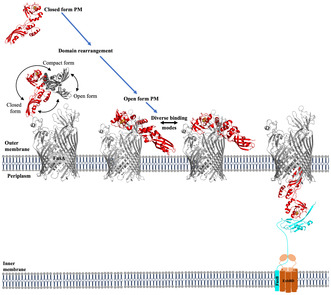

**Abbreviations**
AtFd2: *Arabidopsis* ferredoxin
CSP: chemical shift perturbation
IDTD: intrinsically disordered translocation domain
PM: pectocin M
PM1: pectocin M1
PM1_fd_
: ferredoxin domain of pectocin M1
PM1_full_
: full‐length PM1
PM2: pectocin M2
PMF: proton motive force

*Pectobacterium carotovorum* ranks among the top 10 plant pathogenic bacteria due to its significant destructive effects on agricultural produce throughout the cultivation, storage, and transportation stages [1]. Various control methods such as copper‐based pesticides [2, 3, 4] and antibiotics [5] have been developed to protect agricultural products from infection by this bacterium. However, many pathogenic bacteria have evolved resistance to copper‐based pesticides and bactericides [6]. Hence, focus has shifted toward biologically derived strategies, in particular, environmentally friendly biological agents such as bacteriocins and bacteriophages [6, 7].

Bacteriocins possess high target specificity and ease of expression in plants, making them advantageous in both clinical and agricultural contexts as they are likely to cause minimal microbiome disruption [8]. Treatments using bacteriocins, whether transgenically expressed in plants [8, 9, 10, 11] or applied directly [8, 12, 13], have resulted in strong resistance against phytopathogenic bacteria. Furthermore, bacteriocins are considered to be safe for humans, animals, and non‐targeted bacterial species, with some approved for use in food preservation [11]. Given these benefits, bacteriocins deserve attention for their potential applications. Recently, pectocins, bacteriocins produced by *P. carotovorum* strains, have been shown to eliminate phylogenetically related bacteria [14, 15, 16, 17]; however, the mechanisms by which pectocins are taken up into a target cell are not fully comprehended. Pectocin M1 (PM1), a member of the M‐class bacteriocin family that includes colicin M from *Escherichia coli* [15, 17], exhibits strong growth inhibition against *Pectobacterium* ssp. M‐class bacteriocins exert their lethal effects through enzymatic degradation of a peptidoglycan substrate, lipid‐II [15, 18, 19, 20, 21, 22], resulting in cell lysis and death [23]. They are taken up through a TonB‐dependent receptor and use the TonB‐ExbB‐ExbD machinery, driven by the proton motive force (PMF), to facilitate transportation across the outer membrane of the target cell [24, 25].

In general, M‐class bacteriocins including colicin M possess an N‐terminal intrinsically disordered translocation domain (IDTD) [20, 21, 26] that directly binds to TonB. However, PM1 and PM2 do not have an IDTD domain, but instead contain a globular plant‐type ferredoxin domain at the N terminus [14, 16, 17]. Studies have shown that the TonB‐dependent receptor FusA in the ferredoxin uptake system of *Pectobacterium* ssp. is used for translocation of PMs into cells [16, 27]. Relative to the IDTD, plant‐type ferredoxin is smaller than the plug domain of FusA that occludes the FusA lumen; thus, it can directly interact with the TonB‐like protein FusB, allowing its transport into cells via the TonB‐ExbB‐ExbD machinery [28]. Differing from plant‐type ferredoxin, the dimensions of reported PM2 structures are larger than the FusA plug domain [14]. Consequently, PMs must undergo essential conformational changes to reorient their domains into an extended conformation, enabling them to traverse the lumen of the receptor FusA and enter the periplasm intact. Interactions between the ferredoxin domain of PM1 and FusA extracellular loops have been investigated by NMR [16, 27] and site‐directed mutagenesis [27]; however, the mechanism underlying PM uptake into target cells via the ferredoxin uptake system, especially the initial step of import, remains unclear due to the lack of an atomic resolution structure model of full‐length PM1.

In this study, we have determined the X‐ray structure of full‐length PM1 at a resolution of 2.04 Å, unveiling a previously undocumented ‘closed’ conformation of PMs. Comparison of the distinct domain arrangements in PM1 and PM2 highlights differences in the orientation of conserved amino acids in the active site of the catalytic domain that are correlated with catalytic activity against *Pectobacterium* ssp. Analysis of the diverse domain arrangements in PMs, including our new PM1 structure, reveals the structural flexibility of PMs and how binding to the FusA receptor is enhanced in the initial step of PM uptake. Based on our full‐length structure of PM1 and published chemical shift perturbation (CSP) data on the ferredoxin domain of PM1 [16], we use HADDOCK modeling of FusA with the ferredoxin domain only (FusA:PM1_fd_) and FusA with full‐length PM1 (FusA:PM1_full_) to investigate the interface interactions, optimum conformation, and transient binding of PMs to the FusA receptor during the initial uptake step.

## Materials and methods

### Bacteria strains and plasmids

The target gene sequence encoding PM1 from *P. carotovorum* was determined from the complete genome of *P. carotovorum* subsp. *carotovorum* strain PC1 (NCBI reference: NC_012917). Initially, the gene encoding PM1 was synthesized and fused into pET28a with a His6‐tagged and TEV (tobacco etch virus) protease cleavage sequence at the N terminus (Genscript, Tokyo, Japan); despite optimization of expression conditions, however, no protein was expressed from the plasmid. Consequently, a new recombinant plasmid lacking purification tags was constructed by Gibson assembly using Gibson Assembly Master Mix (New England Biolabs, Ipswich, MA, USA). The polymerase chain reaction primers for amplifying the full‐length PM1 gene and pET28a plasmid were forward_PM1, TACCATGGCGACCTATAAAATCAAGG; reverse_PM1, CGGATCCTTACAGGCGCTGACCTC; forward_pET28, GCCTGTAAGGATCCGAATTCGAGCTC; and reverse_pET28, ATAGGTCGCCATGGTATATCTCCTTCTTAAAG. The resulting construct expressed sufficient PM1 for the structural study.

To create the recombinant plasmid for FusB production, the gene encoding FusB from *P. carotovorum* was identified from complete genome of *P. carotovorum* (NCBI Reference Sequence: WP_138254891). The gene segment encoding the C‐terminal domain of FusB (residues 224–324) was synthesized and inserted into the pET28a vector with the His6‐tagged and TEV protease cleavage sequence at the N terminus (Genscript).

### Expression and purification

PM1 was expressed in *E. coli* BL21(DE3) pLysS host (Invitrogen, Waltham, MA, USA) due to the tight control needed for expression of toxic proteins. Host cells carrying the expression plasmid were cultured in LB broth containing 50 μg·mL^−1^ of kanamycin and 32 μg·mL^−1^ of chloramphenicol. Cell growth was intermittently monitored by measuring optical density at a wavelength of 600 nm (OD_600_). Protein expression was induced at an OD_600_ of 0.6 by adding isopropyl β‐D‐1‐thiogalactopyranoside at a final concentration of 0.5 mm, and growth was continued at 28 °C for 6 h. After centrifugation, the cells were resuspended in 50 mm Tris–HCl pH 7.5, 20 mm NaCl, 0.1 mm PMSF and disrupted by sonication. The resulting lysate was centrifuged at 4 °C for 1 h at 131 656 ***g***, and the supernatant was loaded onto a Cellufine‐A200 anion exchanged open column (CELLUFINE®, Tokyo, Japan) pre‐equilibrated in 50 mm Tris pH 7.5, 20 mm NaCl. The column was washed successively using 10 column volumes (CV) of buffers containing NaCl at concentrations of 20, 50, and 100 mm. Subsequently, the target protein, identified by its distinctive red color, was eluted with 10 CV of buffer containing 500 mm NaCl.

Fractions containing PM1 were pooled and dialyzed overnight against 50 mm Tris–HCl pH 7.5, 20 mm NaCl and then applied to a 5‐mL Hitrap Q HP column (Cytiva, Washington, DC, USA), pre‐equilibrated with 50 mL of 20 mm Tris–HCl pH 7.5. The bound protein was eluted with a linear gradient of 0–1000 mm NaCl in the same buffer. Fractions containing PM1 were treated with ammonium sulfate, added at a final concentration of 40%. After centrifugation (4 °C, 20 min, 7 446 ***g***), the supernatant was loaded onto a Phenyl Sepharose column (GE Healthcare, Chicago, IL, USA). PM1 was eluted with a linear gradient of 40%–0% ammonium sulfate, and red fractions were pooled and concentrated using an Amicon Ultra‐15 Centrifugal Filter Unit (10 kDa MWCO: Millipore, Burlington, MA, USA).

The concentrated PM1 sample was further purified by size exclusion chromatography on a HiLoad® 16/600 Superdex® 75 pg column (Cytiva) equilibrated in 50 mm Tris pH 7.5, 50 mm NaCl. The fractions were analyzed by SDS/PAGE, together with aliquots of concentrated PM1 before injection and fraction 14 that had been heated for 1 min at 90 °C. PM1 was concentrated to 20 mg·mL^−1^ using a 10‐kDa MWCO Amicon filter (Millipore) and stored at −80 °C until crystallization. The absorption features of a 50‐fold dilution solution of purified PM1 at a wavelength of 300–650 nm were determined by using a V‐630 UV–Vis Spectrophotometer (Jasco, Tokyo, Japan).

To prepare PM1 for isothermal titration calorimetry (ITC) measurement, the expression and purification of this protein was performed in the similar way to that for structural study, with the exception of buffer compositions in the gel filtration chromatography. Briefly, PM1 was purified by a HiLoad® 16/600 Superdex® 75 pg column (Cytiva) equilibrated in 20 mm sodium phosphate buffer pH 7.5, and 150 mm NaCl. The purified PM1 was concentrated to 17.60 mg·mL^−1^.

To purify FusB for ITC measurement, *E. coli* BL21(DE3) host cells carrying the recombinant plasmid was cultured in LB broth containing 50 μg·mL^−1^ kanamycin. The cells were cultured until the OD_600_ reach to 0.6. The resulting culture was induced by IPTG at the final concentration of 0.5 mm followed by continued growth at 20 °C for 16 h. After centrifugation, the cells were resuspended in lysis buffer containing 50 mm Tris–HCl pH 7.5, 500 mm NaCl, 0.5 mg·mL^−1^ lysozyme, and 0.1 mm PMSF. The cell resuspension was then disrupted by sonication. The resulting lysate was centrifuged at 4°C for 1 h at 32 000 rpm, and the supernatant was collected and then loaded onto the nickel open tubular column pre‐equilibrated in buffer containing 50 mm Tris pH 7.5, 500 mm NaCl. The column was washed using 10 CV of buffers containing imidazole at concentrations of 10, 20, 50, and 250 mm. The sample eluted with buffer containing 250 mm imidazole was collected and then dialyzed against 50 mm Tris–HCl pH 7.5, 500 mm NaCl overnight. The dialyzed sample was treated with TEV protease at 20 °C for 2 h. The TEV protease‐treated sample was loaded onto the nickel column to remove the residual tag. The flowthrough was collected and concentrated using a 10‐kDa MWCO Amicon filter (Millipore). Then, the concentrated sample was further purified by a HiLoad® 16/600 Superdex® 75 pg column (Cytiva) equilibrated in buffer containing 20 mm sodium phosphate buffer pH 7.5, and 150 mm NaCl. Subsequently, the eluted fractions containing FusB were concentrated to 0.28 mg·mL^−1^.

### Crystallization and diffraction data collection

Crystallization trials were performed by using the sitting‐drop vapor‐diffusion method at a PM1 concentration of 20 mg·mL^−1^. Crystallization droplets were generated by mosquito LCP technology (SPT Labtch, Royston, UK) using 100 nL of 20 mg·mL^−1^ PM1 and 100 nL of reservoir solution from commercial screening kits (∼ 400 conditions) at 4 and 20 °C. A crystal was observed in Index™ 59 (0.02 m magnesium chloride hexahydrate, 0.1 m HEPES pH 7.5, 22% w/v polyacrylic acid sodium salt [PAS] 5100; Hampton Research, Aliso Viejo, CA, USA) at 20 °C. Further optimization using the hanging‐drop vapor‐diffusion method in 0.1 m HEPES pH 7.5 and 19.2% w/v PAS 5100 resulted in the formation of brown crystals with rod and plate clusters.

The crystals were looped and cryoprotected by soaking for a few seconds in crystallization solution plus 20% glycerol before cryocooling in liquid N_2_. The X‐ray diffraction experiment was performed at SPring‐8 using beamline BL44XU.

### Structure solution and refinement

The datasets were processed by xds software [29]. The best dataset, which diffracted at 2.04 Å resolution, was solved by molecular replacement using the structure of PM2 (PDB ID: 4n58) [14] and Phaser from the phenix package [30, 31]. An initial model was built using the autobuild program in phenix. The model was built further and refined interactively using coot software [32] and phenix.refine [33] at 2.04 Å resolution. The final refined coordinate and structure factors of PM1 have been deposited in the Protein Data Bank under the accession number 8jc1. Since the chain B provided the clearest electron density of helical linker region, we select chain B as a representative of PM1 structure for further analysis.

### Interdomain interaction analysis

The coordinates of chain B from the PM1 crystal structure were used for interdomain interaction analysis using the DIMPLOT program in the Ligplot^+^ suite [34]. The ferredoxin domain (residues 2–94) was designated as domain A; the remaining regions (residues 95–268), including the catalytic domain and linker, were defined as domain B.

### Domain movement and rotational angle analysis

Rotational motions and angles between the catalytic domains of different conformations of pectocins were analyzed using dyndom [35]. Chain A from both PM2 structures (PDB ID: 4n58 and 4n59) [14] and chain B of PM1 were selected for this assessment. Pectocin coordinates, including open‐form PM2 (PDB ID: 4n58), compact‐form PM2 (PDB ID: 4n59), and closed‐form PM1, have been submitted to the DynDom web server (https://dyndom.cmp.uea.ac.uk/dyndom/runDyndom.jsp) for analysis.

### HADDOCK simulation

Simulated docking models of FusA (PDB ID: 4zgv) [16] and the ferredoxin domain of PM1 were simulated using haddock 2.4 [36] by submitting the coordinates to the HADDOCK web server (https://wenmr.science.uu.nl/haddock2.4/). The interacting passive residues within the extracellular loops of FusA were assigned based on previous data [16]. Active residues in the ferredoxin domain were defined based on the criteria described in Section Docking models of the complex between PM1 and FusA. During the simulation, 200 structures were selected for the final refinement and then clustered into 10 groups based on the Fraction of Common Contacts of 0.60. The parameters in the docking were based on the default settings of the server, with the exception of a 25% removal of restraints (Data S1). The N and C terminus for all proteins were treated as charged and uncharged, respectively. Detailed statistics for each cluster resulting from the docking simulation are presented in Table S1.

### ITC measurement

For ITC measurement, proteins were prepared in 20 mm sodium phosphate buffer pH 7.5 and 150 mm NaCl. Measurement was performed using a VP‐ITC instrument (Malvern Panalytical Ltd., Malvern, UK) at 25 °C with stirring speed of 307 rpm. The syringe contained 480 μm of PM was titrated into 20 μm FusB in the ITC cell.

## Results

### Structure determination

**Fig. 1:**
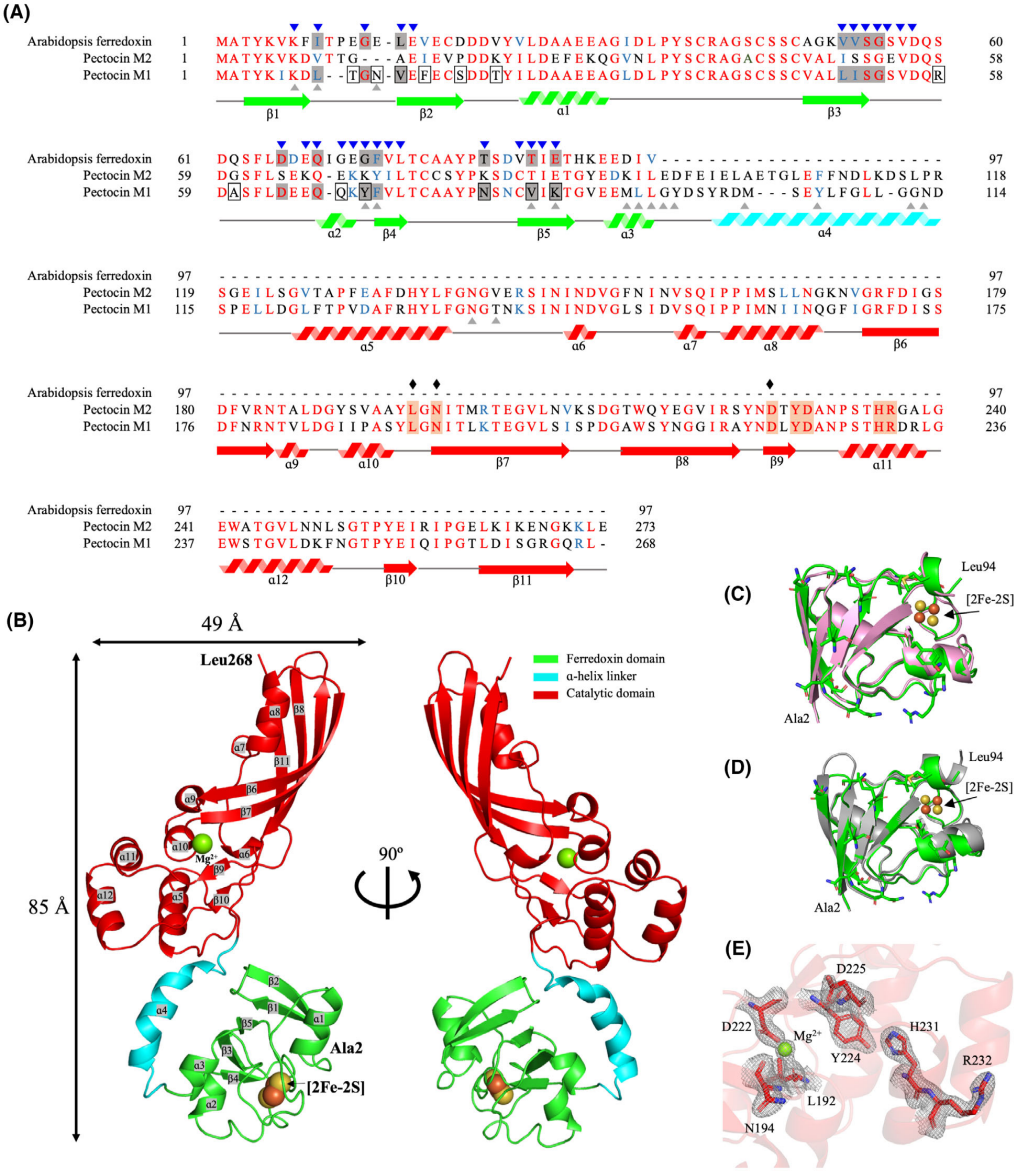
Comparison of PM1 with other ferredoxin domain‐containing proteins. (A) Multiple sequence alignment of PM1, PM2, and AtFd2. Identical residues are in red, similar residues in blue, and unique residues in black. Colored arrows and zigzags denote the ferredoxin domain (green), ɑ‐helix linker (cyan), and catalytic domain (red). Blue arrowheads indicate residues with CSP values of > 0.02 p.p.m. in a previous FusA–PM1_fd_ NMR titration (1 : 1 molar ratio) [16]. A gray background indicates active residues in HADDOCK docking; black boxes highlight amino acids unique to the PM1 ferredoxin domain; gray arrowheads indicate amino acids of PM1 involved in interdomain interactions as analyzed by Ligplot^+^. Key active‐site residues of the catalytic domain are highlighted with an orange background; black diamonds indicate amino acids that interact with the Mg^2+^ cofactor. (B) Schematic depicting the crystal structure of PM1 observed in the *P*2_1_ space group. The ferredoxin domain is green, α‐helix linker cyan, and cytotoxic domain red. Spheres represent the [2Fe‐2S] cluster. (C) Schematic showing the ferredoxin domain of PM1 (green) superimposed with that of PM2 (pink; PDB ID: 4n58) (backbone r.m.s.d. = 0.529 Å, PM1 residues = 2–94, PM2 residues = 2–94). (D) Schematic illustrating the ferredoxin domain of PM1 (green) aligned with that of AtFd2 (gray; PDB ID: 4zho) (backbone r.m.s.d. = 0.678 Å, PM1 residues = 2–94, AtFd2 residues = 2–98). Unique amino acids in the ferredoxin domain of PM1 are represented by sticks (black boxes in (A)). (E) Schematic diagram of the active site of catalytic domain with electron density map.

The PM1 protein, including the ferredoxin domain (residues 1–95), α‐helix linker (96–116), and catalytic domain (117–268), contains 268 amino acids with a molecular mass of 29.3 kDa (Fig. 1A). The gel filtration profile of the purified sample revealed a predominant peak at 280 and 422 nm, indicative of the presence of a [2Fe‐2S] cluster in ferredoxin domain (Fig. S1A). SDS/PAGE indicated a purity of more than 95%, but the observation of two bands in non‐heated samples and one band in heated samples showed that PM1 adopts multiple conformations in solution (Fig. S1B). The absorption spectrum of purified PM1 showed peaks at 330, 423, and 466 nm (Fig. S1C), consistent with that of other pectocins and ferredoxins containing a [2Fe‐2S] cluster [17, 37, 38].

Initial crystallization trials yielded small rod‐shaped crystals of PM1 after 2 days in the condition of 0.02 m magnesium chloride hexahydrate, 0.1 m HEPES pH7.5, and 22% w/v PAS 5100 at 20 °C (Fig. S2A). Both rod and plate‐shaped crystals were observed in the optimized condition of 0.1 m HEPES pH 7.5 and 19.2% w/v PAS 5100 via the hanging‐drop vapor‐diffusion method (Fig. S2B). One rod‐shaped crystal diffracted well, providing the monoclinic space group of *P*2_1_ with unit cell parameters of *a* = 66.28 Å, *b* = 138.55 Å, *c* = 68.95 Å, and β = 94.18°. The estimated number of molecules in the crystallographic asymmetric unit was four with a Matthews coefficient (*V*_M_) of 2.63 Å^3^·Da^−1^ and solvent content of 53.3%.

Molecular replacement using the full‐length PM2 structure as a template failed. Thus, the catalytic and ferredoxin domain of PM2 were independently used as templates (PDB ID: 4n58). The initial model from AutoBuild package was further refined at 2.04 Å resolution. The crystallographic statistics are summarized in Table 1. The final model contains four PM1 molecules in the crystallographic asymmetric unit. Each monomer, comprising an N‐terminal ferredoxin domain and a C‐terminal catalytic domain interconnected by an α‐helix, is in principle the same (Fig. S4); therefore, the model of chain B (Fig. 1B) has been used as a representative in subsequent analyses.

**Table 1:** Data collection and refinement statistics.

Data collection	PM1
Beamline	Spring‐8 BL44XU
Wavelength (Å)	0.9000
Resolution (Å)	40.27–2.04 (2.12–2.04)^a^
Space group	*P*2_1_
Cell dimensions	
*a*, *b*, *c* (Å)	66.3, 138.5, 67.0
ɑ, β, 𝛾 (°)	90, 94.2, 90
Total reflections	275 746 (28 529)^a^
Unique reflections	78 357 (7963)^a^
Mean *I*/sigma (*I*)	9.73 (1.61)^a^
Completeness (%)	98.6 (91.2)^a^
*CC(1/2)*	0.99 (0.77)^a^
Multiplicity	3.5 (3.6)^a^
Wilson plot *B*‐value (Å^2^)	38.86
*R*‐meas^b^	0.053 (0.794)^a^
Refinement	
Reflections used in refinement	77 351 (7126)^a^
Reflections used for *R‐*free	1976 (170)^a^
*R‐*work*/R‐*free^c^	0.195/0.239
No. of non‐hydrogen atoms	8765
Protein	8158
Ligand/ion	67
Water	540
Protein residues	1068
Average *B*‐factor (Å^2^)	46.9
Macromolecules	47.0
Ligands	60.6
Solvent	45.0
Ramachandran plot statistics	
Favored (%)	95.6
Allowed (%)	4.0
Outliers (%)	0.4
Ramachandran *Z*‐score	−1.03
R. m. s. deviations	
Bond lengths (Å)	0.007
Bond angles (°)	0.85
R. m. s. *Z*‐scores	
Bong lengths	0.39
Bong angles	0.60

^a^ Statistics in parentheses represent values calculated from the highest resolution shell

^b^ *R*_meas_ *= ∑*_*hkl*_ *∑*_*i*_ *|I*_*i*_ *(hkl)‐ < I*_*i*_ *(hkl) > |/∑*_*hkl*_ *∑*_*i*_ *I*_*i*_ *(hkl)*, where *i* = number of observations of a given reflection; *I(hkl)* = average intensity of the *i* observations. *R*_free_ was calculated using a 5% fraction of randomly selected reflections evaluated from refinement. Highest resolution shells are given in parentheses

^c^ *R*_work_ = *∑*_*hkl*_ *||F*_obs_ *|‐|F*_calc_ *||/∑*_*hkl*_ *|F*_obs_ *|*, *R*_free_ was calculated for 5% randomly selected test sets that were not used in the refinement.

### Structure of PM1

The overall structure of PM1 has dimensions of 49 Å (width) and 85 Å (length), and is clearly larger than the FusA plug domain. Each domain of PM1 looks similar to its counterpart in the published PM2 structures (PDB ID: 4n58 and 4n59); however, the three‐dimensional arrangement of these domains in PM1 differs from that in PM2.

**Fig. 2:**
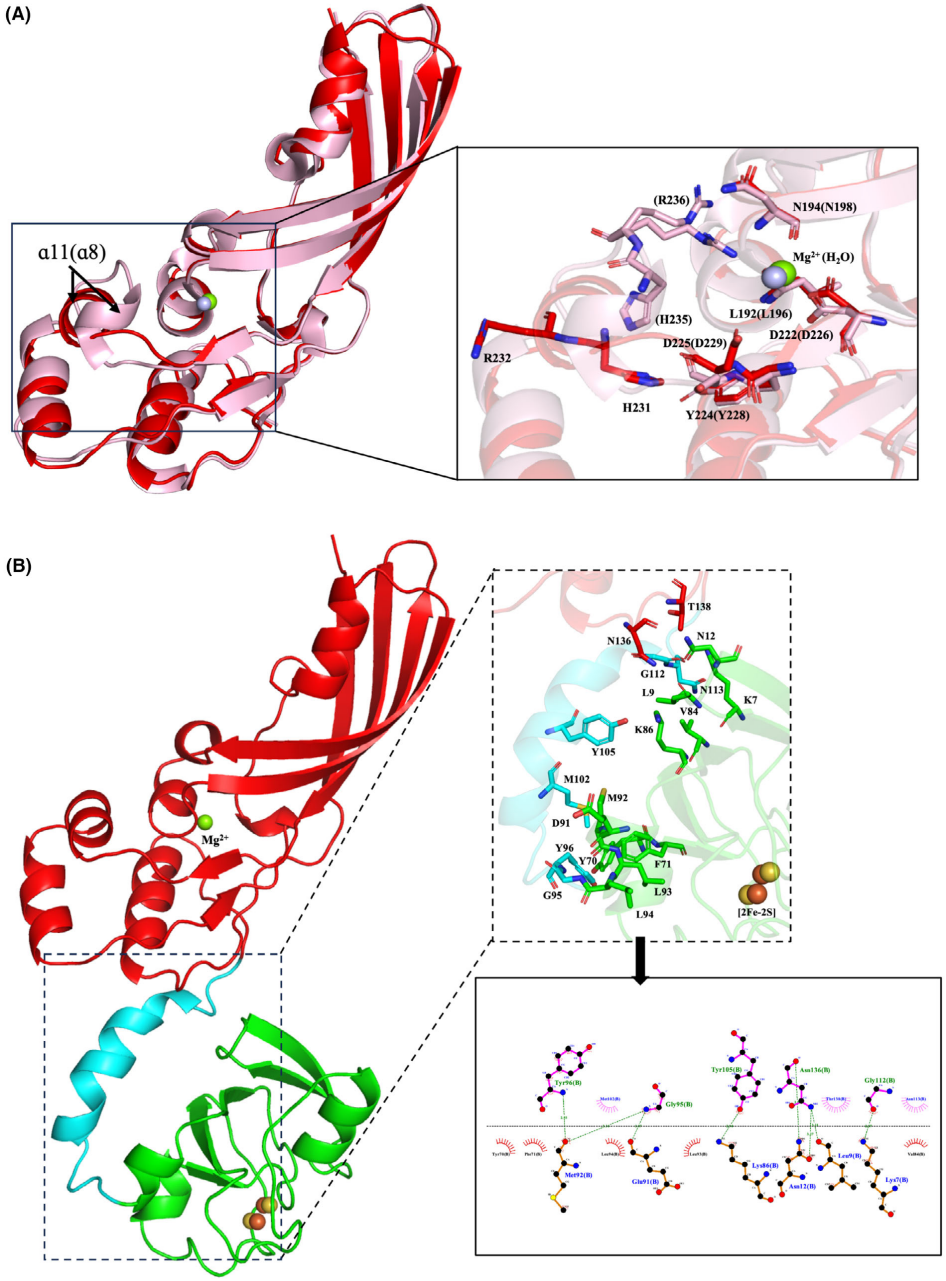
Molecular insights into PM1 interdomain interactions and the active site. (A) Schematic alignment of the cytotoxic domains of PM1 (red) and PM2 (pink; PDB ID: 4n58) (backbone r.m.s.d. = 0.733 Å, PM1 residues = 120–268, PM2 residues = 124–271). The magnified view in the black panel focuses on the active site in PM1 and PM2, displaying key conserved residues as sticks with PM2 residues given in brackets. Mg^2+^ cofactor in PM1 and water molecule in PM2 are represented as green and light blue spheres, respectively. (B) Analysis of key amino acids of PM1 involved in interdomain interactions by LigPlot^+^, highlighting residues in the ferredoxin domain in green, others in cyan and red as shown in the black dashed box. The magnified view in the black panel shows the interdomain interaction region, displaying key amino acids represented as sticks and the [2Fe‐2S] cluster as spheres.

Multiple sequence alignment reveals that the ferredoxin domain of PM1 possesses 13 unique residues (Fig. 1A, boxes), which are located on the back side of the domain away from the [2Fe‐2S] cluster. This side is thought to be crucial for binding to FusA [16]. The ferredoxin domain itself shows 0.529 Å of r.m.s.d. based on Cα carbons and 60.6% of sequence identity to that of PM2 (PDB ID: 4n58, Fig. 1C) [14], and 0.678 Å and 63.9%, respectively, to that of *Arabidopsis thaliana* Fd2 (AtFd2) (PDB ID: 4zho, Fig. 1D), suggesting that differences exist only in the surface structure attributed to side chains. The PM1 catalytic domain adopts an elongated structural feature of mixed α‐helix/β‐sheet components (Figs 1B and 2A), commonly found in M‐class bacteriocins [14, 20, 21, 26]. The backbone structure of the catalytic domain in PM1 also shows high similarity to that of PM2 (PDB ID: 4n58) with an r.m.s.d. of 0.733 Å based on Cα atoms (Fig. 2B).

A strong peak in the electron density map is found in the active site of catalytic domain, similar to observations for PM2 [14], pyocin M (PaeM) [20], and syringacin M (SyrM) [21]. We assigned this as an Mg^2+^ ion because it fitted perfectly in this map with better values of *B*‐factor and ligand coordination (Fig. 1E) [39], consistent with the catalytic performance of PM1 in the presence of Mg^2+^ ions [15]. Amino acid residues conserved in the active sites (Figs 1A and 2A, highlighted with an orange background) are similarly oriented, particularly metal‐coordinated residues (Fig. 1A, black diamonds). A notable difference is the position of the α11 helix in PM1. This helix aligns in parallel with the neighboring α5 and α12 helices in PM1, whereas the corresponding helix positions are perpendicular in PM2, leading to a different orientation of the conserved His231 and Arg232 (PM1) and corresponding His235 and Arg236 (PM2) residues (Fig. 2A, right). In the PM1 structure, these two residues are situated apart from other conserved residues, opening up the active site. Conversely, in the PM2 structures, these residues (His235 and Arg236) are directed toward two other conserved residues in the active site, Asn198 and Asp226 (PM2), which form hydrogen bonds with a water molecule and thereby prevent substrate from accessing the active site. The resulting orientation of His235 and Arg236 of PM2 prevents substrate from accessing the active site as shown in Fig. 2A.

### Domain arrangement and interdomain interaction of PM1

The two dimensions of PM1 obtained from the crystal structure in this work (85 Å × 49 Å) are similar to those determined by DAMMIF model in the SAXS analysis from the previous work (82 Å × 54 Å) [27], suggesting that the PM1 in the closed conformation is the most stable form and predominantly exits in solution. However, the CRYSOL SAXS curve predictions, coupled with ensemble optimization modeling, revealed that PM1, like PM2, is flexible and adopts multiple conformations with maximum dimensions ranging from 70 to 90 Å [27]. These findings support the existence of the domain rearrangement in PM1 between the closed form (the maximum dimension of 85 Å) and these potential intermediate conformations. The domain arrangement found in PM1 is differs completely from that in the two PM2 structures. We call the PM1 domain arrangement the ‘closed’ form, consistent with a previous small‐angle X‐ray scattering (SAXS) study [27]. Conversely, the two structures of PM2 show ‘open’ (PDB ID: 4n58) and ‘compact’ (PDB ID: 4n59) forms, both of which expose their back side of the ferredoxin domain to the solvent [14]. We used Ligplot^+^ to assess interdomain interactions, which revealed key amino acids on this side of the ferredoxin domain (Lys7, Leu9, Asn12, Tyr70, Phe71, Val84, Lys86 Met92, Leu93, Leu94; Fig. 1A, gray arrowheads; Fig. 2B right, stick models) that interact extensively with amino acids in other domains (Gly95, Tyr96, Met102, Tyr105, Gly112, Asn113, Asn136, Thr138; Fig. 2B). Among them, Lys7, Leu9 and Asn136 are conserved between PM1 and PM2, implying that some interdomain interactions are shared in the two protein isoforms.

**Fig. 3:**
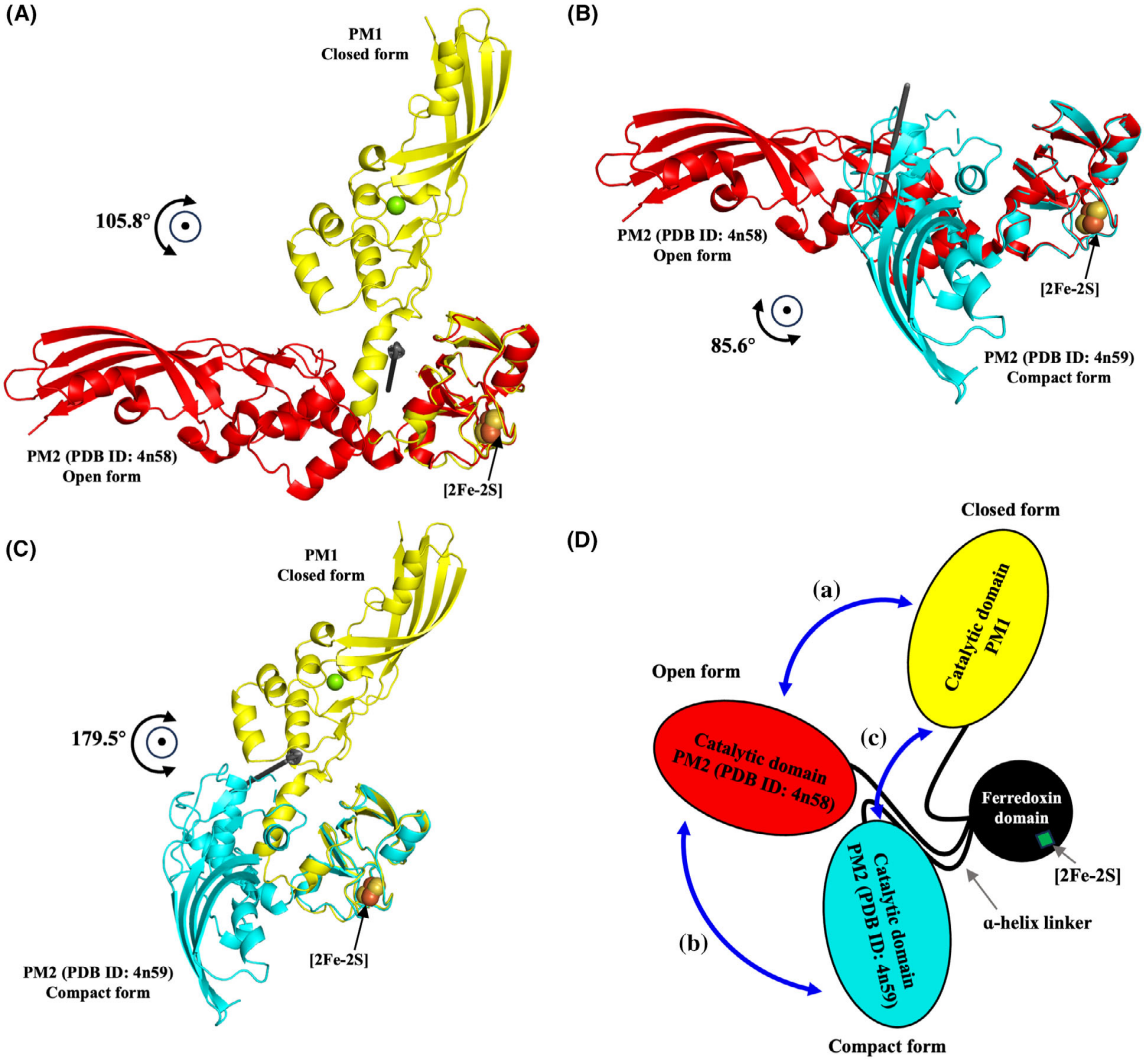
Comparative analysis of domain motions and rotational angles of cytotoxic domains between diverse conformations of PM. Shown are domain movement and rotational angles of the cytotoxic domain via the flexible linker between (A) PM1 and PM2 (PDB ID: 4n58), (B) PM2 (PDB ID: 4n58) and PM2 (PDB ID: 4n59), and (C) PM1 and PM2 (PDB ID: 4n59) calculated by dyndom. Cartoons represent the crystal structure of PM1 (yellow), PM2 (red; PDB ID: 4n58), and PM2 (cyan; PDB ID: 4n59). The [2Fe‐2S] cluster is shown as spheres. (D) Schematic showing the rotational motion between the cytotoxic domain of different conformational pectocins: PM1 (yellow), PM2 (red; PDB ID: 4n58), and PM2 (cyan; PDB ID: 4n59). The ferredoxin domain and the [2Fe‐2S] cluster are represented by black spheres and green squares, respectively; the linker is shown as a black line.

Even for the closed or compact form of pectocin, the molecular dimensions are bigger than the plug domain of FusA, which means that domain rearrangement or structure melting must take place to allow their translocation into the periplasm. To visualize and quantify the varied domain arrangement in PM2 suggested previously by SAXS [27, 40], we calculated the differential rotational angles of the catalytic domains in the three forms of PM structure using the dyndom program [35] as 105.8° (open vs closed form; Fig. 3A), 85.6° (open vs compact; Fig. 3B), and 179.5° (compact vs closed; Fig. 3C). Based on these data, we propose that there is preferred but restricted rotational movement of the catalytic domain via the α‐helix linker, which might be important for the ferredoxin domain to expose its back side to FusA (Fig. 3D, Movie S1).

### Docking models of the complex between PM1 and FusA

**Fig. 4:**
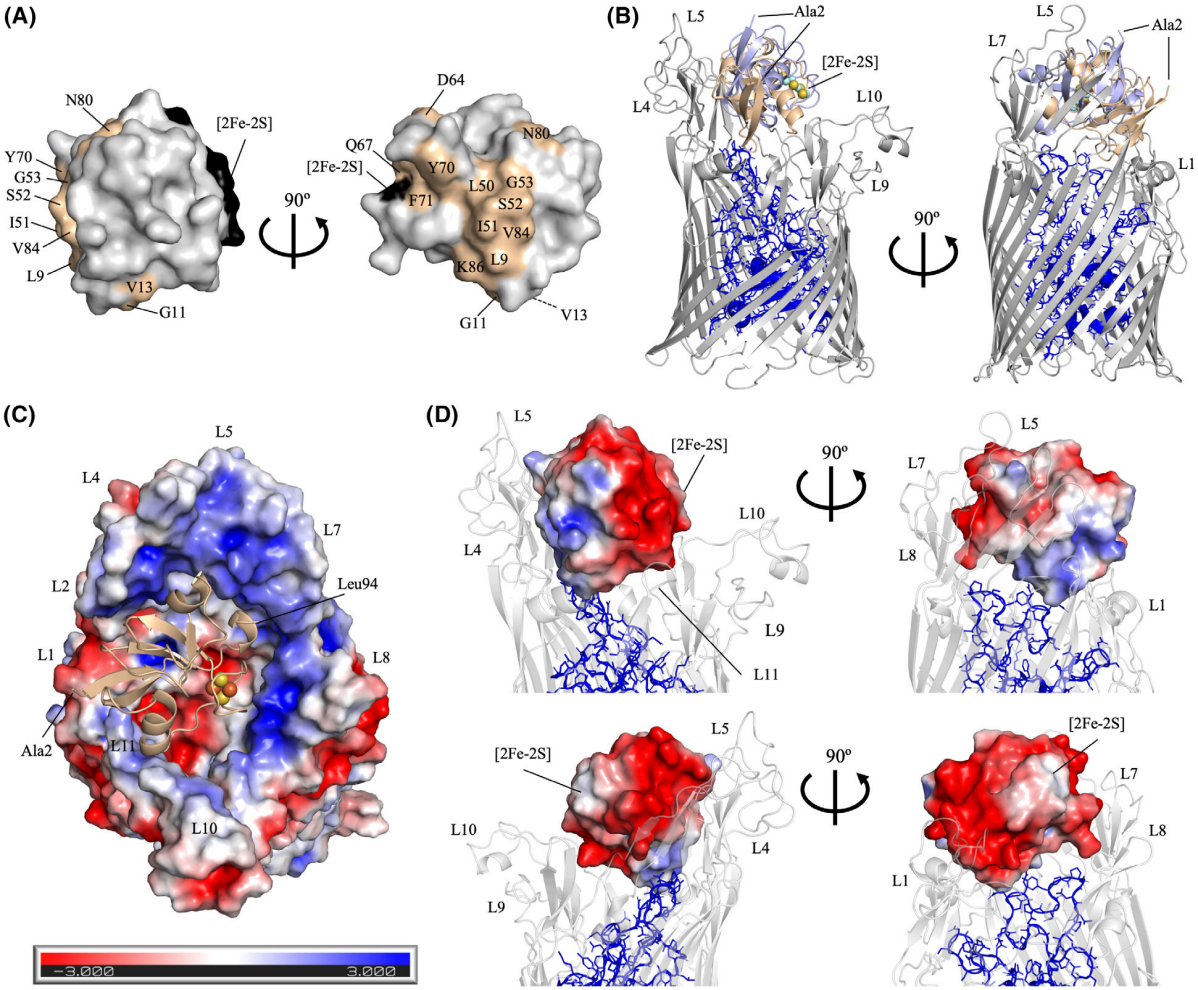
Structural analysis of FusA and PM1_fd_ via HADDOCK simulation. (A) Active residues assigned in the simulation (indicated on a gray background in Fig. 1A) are mapped onto the surface of the 3D structure of PM1_fd_ (wheat color). (B) Crystal structure of FusA (gray) with the top two models of PM1_fd_ superimposed (wheat color, 1^st^ rank; light blue, 2^nd^ rank). The plug domain of FusA is shown as a blue stick and cartoon model; the [2Fe‐2S] cluster is shown as spheres. (C) Top scored model of the FusA–PM1_fd_ complex. FusA is shown as a molecular surface mapped with electrostatic potential; the crystal structure of PM1_fd_ is shown as a wheat cartoon model. (D) Molecular surface mapped with electrostatic potential of PM1_fd_ docked to FusA viewing from the front (top panel) and the back (bottom panel) with the right panel rotated in 90° each, shown as a white cartoon with its plug domain highlighted as a blue cartoon and stick model.

Next, we explored the structure of the PM1–FusA complex using our X‐ray structure of PM1 coupled with a previous NMR‐based interaction study between FusA extracellular loops and the ferredoxin domain of PM1 [16]. First, we mapped residues of the ferredoxin domain of PM1 reported to have a CSP of > 0.02 ppm upon FusA addition at a 1 : 1 molar ratio [16]; (Fig. 1A, residues with a gray background) on the X‐ray structure of PM1 (Fig. 4A, wheat‐colored residues). The complex structure between the ferredoxin domain of PM1 and FusA (FusA:PM1_fd_) was then predicted using the haddock 2.4 program [36]. Below, we describe the two models with the best scores (Data S2 for the 1^st^ and Data S3 for the 2^nd^ model, Table S1). In both predicted models, the ferredoxin domain is bound to the extracellular loops of FusA, exhibiting a similar binding mode through its back side but with a rotational variation of 50.4° (Fig. 4B). Notably, the previously reported docking models of FusA:AtFd2 [16] show a distinct binding mode of AtFd2 (Fig. S3).

In both FusA:PM1_fd_ models, the plug domain and the L1, L4, L5, L7, and L11 loops of FusA accommodate PM1_fd_ (Fig. 4C,D), whereas the plug domain and only three loops of FusA (L4, L5, and L7) host AtFd2 in the FusA:AtFd2 model (Fig. S3C,D). This difference in prediction may be caused by sequence variations in the back side of the ferredoxin domains (Fig. 1A, black boxes). Mapping the electrostatic potentials onto the surface of FusA, PM1_fd_, and AtFd2 clearly explains these preferences in binding (Fig. 4C; Fig. S3C). While the inner walls of FusA (L2, L4, L5, L7, L8, L10 and L11 loops) are positively charged and the plug domain (Fig. 4B,D, blue stick model; and Fig. S3B,D) exhibits a negative charge on the inner floor (Fig. 4C and Fig. S3C), the back sides of PM1_fd_ and AtFd2 are clearly different with more negative charges in AtFd2, and less negative and even positive charges in PM1_fd_.

**Fig. 5:**
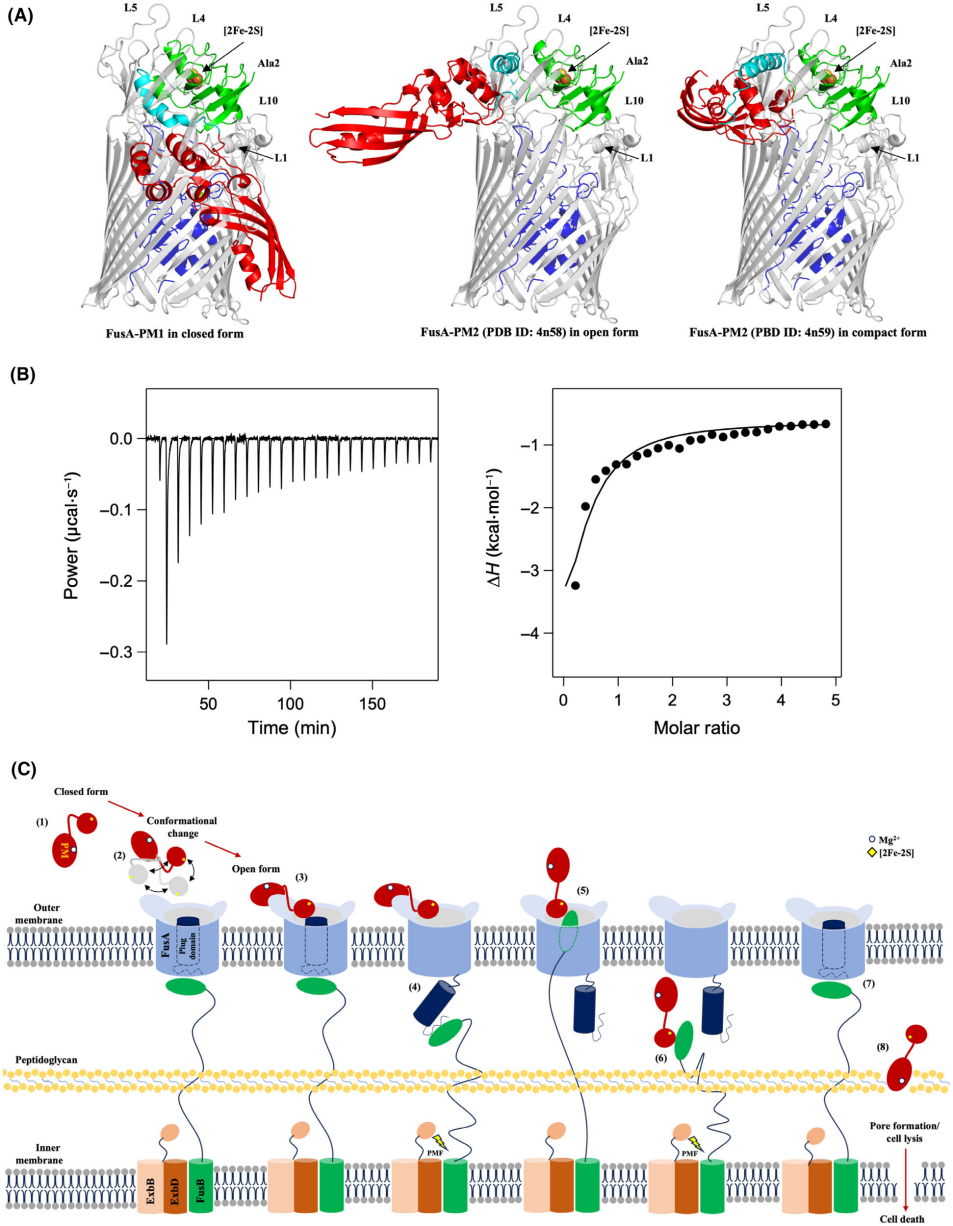
Model of the uptake of intact PM via the receptor FusA into *Pectobacterium* ssp. cells. (A) Structural superimposition of the ferredoxin domain of the FusA–PM1_fd_ complex obtained from HADDOCK docking with that of the different conformations of PM shows that PM forms extensive interactions and collisions with the extracellular loops and plug domain of FusA. FusA and PM are depicted as cartoon models. FusA and its plug domain are colored gray and blue, respectively, while the ferredoxin domain, the helical linker, and the catalytic domain of PM are colored in green, cyan, and red, respectively. (B) ITC thermogram of the tritration of PM1 to FusB obtained at 25 °C under 20 mm sodium phosphate pH 7.5, 150 mm NaCl is shown in left. FusB‐PM1 binding isotherms obtained from the ITC thermogram is shown in right. The calculated values for FusB‐PM1 complex are *K*_d_ = 8.1 μm, Δ*G* = −6.9 kcal·mol^−1^, Δ*H* = −6.0 kcal·mol^−1^, and ‐*T*Δ*S* = −0.9 kcal·mol^−1^. (C) Proposed mechanism of the uptake of intact PM through the receptor FusA into *Pectobacterium* ssp. cells via the ferredoxin uptake system. In the extracellular environment, PM in the closed form (1) reaches the extracellular loops and plug domain of FusA, inducing a conformational change to the open form (2) and exposing the binding surface of PM to the extracellular loops and plug domain of FusA (3). The resultant binding triggers release of the FusA plug domain into the periplasmic space, where it binds to FusB through energy transduced from the proton motive force (PMF) generated by the ExbBD complex (4). Subsequently, FusB enters the FusA lumen and interacts with the ferredoxin domain of PM (5). Using energy from PMF, FusB induces a conformational change of intact PM to an elongated form and then translocates PM into the periplasm (6). The plug domain re‐enters the FusA lumen, restoring FusA and FusB to their resting states (7). Lastly, PM binds to peptidoglycan, initiating digestion of the lipid‐II substrate via its catalytic domain and leading to pore formation, cell lysis, and ultimately cell death (8).

Finally, we superimposed the full‐length PM1 structure and the two PM2 structures on the best‐score FusA:PM1_fd_ model (Fig. 5A). Using the contact program in the CCP4 suite [41], a number of amino acid residues collided with the FusA molecule in all cases (Table S2). The degree of collision was highest for the closed conformation, intermediate for the compact form, and lowest for the open conformation when simply superimposed.

### Thermodynamic changes upon complex formation between FusB and PM1

The binding isotherm (Fig. 5B) revealed that the strong binding interaction between FusB and PM1 is driven mainly by enthalpy, with contributions from entropy as well, with *K*_d_ = 8.1 μm, Δ*G* = −6.9 kcal·mol^−1^, Δ*H* = −6.0 kcal·mol^−1^, and −*T*Δ*S* = −0.9 kcal·mol^−1^. These parameters suggest that the complex formation of FusB‐PM1 is a thermodynamically favored and spontaneously occurred. The large negative enthalpy contribution in complex formation between FusB and PM1 may reflect the release of heat following direct molecular binding at the protein surfaces.

## Discussion

Comparison of the catalytic domain of PM1 with that of PM2 (PDB ID: 4n58) revealed that the two domains had nearly identical structures due to high sequence similarity (> 60%). However, a difference in orientation between the α11 helix in PM1 and the corresponding α8 helix in PM2 alters the relative orientations of the conserved His231 (His235 in PM2 numbering) and Arg232 (Arg236) residues on these helices (Fig. 2A), creating a much more open active‐site cavity in PM1 that enhances the accessibility of peptidoglycan lipid‐II intermediates. A conserved arginine residue is similarly positioned outside the active site in the homologous bacteriocins pyocin M and Syringacin M, also resulting in open active‐site cavities [20, 21]. This observation suggests that there is high flexibility in these regions within the M‐class bacteriocin family, potentially aiding in lipid‐II coordination and stabilization of the pyrophosphate group near key catalytic residues [14]. Although the catalytic mechanism of M‐class bacteriocins toward lipid‐II substrates remains unclear, we suggest that the PM1 structure identified in this work represents a highly active‐site conformation, while the previous PM2 structures represent a less active state, which correlates with their catalytic performance toward *Pectobacterium* ssp. [14, 17].

Owing to the lack of an available PM1 structure, a previous model of the uptake of PM1 through FusA was proposed based on NMR spectroscopy and subsequent site‐directed mutagenesis [27]. Our newly determined X‐ray structure of full‐length PM1 allows us to develop this model further. In the PM1 structure, the ferredoxin domain and colicin M‐like cytotoxic domain are uniquely interconnected by a flexible helix linker in a ‘closed’ conformation. HADDOCK‐simulated models of FusA:PM1_full_ complexes based on the FusA:PM1_fd_ complex (Fig. 5A, left) revealed extensive collision between FusA and PM1 in the closed conformation. We therefore performed docking between FusA and full‐length PM with the previously documented open and compact forms of PM2 (Fig. 5A, middle and right). However, none of three docked models looks feasible because of collision; thus, domain rearrangement is clearly required for productive complex formation between PM and the extracellular loops of FusA. This domain rearrangement may involve a partial melt to facilitate translocation of PM with its additional catalytic domain relative to plant‐type ferredoxin. Our predicted model of FusA:PM1_fd_ shows clear differences from the previously predicted FusA:AtFd2 model in terms of orientation and position of the ferredoxin domain [16] (Fig. 4 and Fig. S3). Differences in the sequence of the back side of plant ferredoxin and ferredoxin domain of PMs, which would impact binding energies and electrostatic potential between FusA and the ferredoxin domain, may be central to the domain rearrangement function, suggesting that these interactions are crucial for facilitating the binding of PMs to FusA in the initial transportation step of intact PM through the FusA lumen.

Figure 5C shows a model of PM translocation via a partial melt of structure at the domain level. In this model, (1) PM adopts the closed conformation for stability and to prevent the binding interface on the ferredoxin domain interacting with other molecules in the extracellular environment. (2) When the closed‐form PM reaches the extracellular loops of FusA embedded in the outer membrane of the target cell, it fluctuates among its diverse conformations via movement of the catalytic domain and α‐helix linker to produce the open conformation, (3) enabling the ferredoxin domain to bind extensively to the extracellular loops and plug domain of FusA, as analyzed using the dyndom program [35]. (4) Binding of PM to the plug domain of FusA causes dislocation of the plug domain to the periplasm, where it is bound by FusB through energy transduced from PMF generated by the ExbBD complex.

To pass through the outer membrane, intact PM needs to adopt an elongated conformation that closely fits the dimensions of the FusA lumen. Because the dimensions are so similar, it seems unlikely that PM passively diffuses through this pore. Also, ITC measurement confirmed the direct interaction between FusB and pectocin M1 in solution for energizing the import process, demonstrating binding kinetics and affinity similar to those observed for the FusB‐ferredoxin interaction [28]. Therefore, we suggest that, (5) when FusB inserted into the FusA lumen interacts with the ferredoxin domain of PM bound to the FusA extracellular loops, it triggers a change in PM to the elongated conformation. This process probably relies on (6) a subsequent step driven by PMF, which would be essential for pulling the elongated PM through the FusA lumen. In the next step, (7) PM is released into the periplasm, allowing the plug domain to re‐enter the FusA lumen, which returns FusA and FusB to their resting states. (8) Lastly, PM reaches the peptidoglycan lipid‐II intermediates in the periplasm and digests them, leading to pore formation, cell lysis, and ultimately, cell death.

In summary, we have described the structural and dynamic characteristics of PM1, a bacteriocin from *P. carotovorum*, focusing on its interaction with the outer membrane receptor FusA in the initial step of PM uptake via the ferredoxin uptake system. The crystal structure of full‐length PM1 reveals a previously undocumented closed domain arrangement, stabilized by extensive interdomain interactions. This structure, together with docking models simulated in this study, provides insights into the structure‐based translocation mechanism of PM1 during the initial steps of its importation into the periplasmic space of susceptible cells, highlighting its parasitization of the FusA receptor of the ferredoxin uptake system. Comparison with AtFd2 structure highlights structural similarities but also differences in the electrostatic surface properties on the back side of the ferredoxin domains. These differential electrostatic potentials may explain why structural differences not in the main chain but in the side chains affect the preference of binding position and orientation of PM1_fd_ to FusA in the docking models. Collectively, our findings provide insights into the structure‐based mechanism of PM1 during the initial steps of its importation into periplasmic space of susceptible cells by parasitizing receptor FusA of the ferredoxin uptake system.

## Conflict of interest

The authors declare no conflict of interest.

### Peer review

The peer review history for this article is available at https://www.webofscience.com/api/gateway/wos/peer‐review/10.1002/2211‐5463.13874.

## Author contributions

GK supervised the study; GK and NJ designed experiments; HT and NJ performed crystallographic experiments; YL and Y‐HL performed ITC measurement and analysis; GK, HT, and NJ analyzed data and wrote the manuscript; All authors made manuscript revisions.

## Supporting information


**Fig. S1.** Validation of PM1 purification.
**Fig. S2.** Pectocin M1 crystallization and crystal morphology.
**Fig. S3.** Structural analysis of the FusA–AtFd2 complex obtained via HADDOCK docking simulation.
**Fig. S4.** Superimposed models of the catalytic domain of four PM1 molecules in the crystallographic asymmetric unit (residues = 135–267).
**Table S1.** HADDOCK docking statistics.
**Table S2.** Analysis of collisions of amino acid residues in FusA–Pectocin M model complexes.

**Data S1.** The parameters used in HADDOCK docking of the FusA‐PM1_fd_ complex.

**Data S2.** The structural coordinates of 1^st^‐rank HADDOCK‐generated docking model of the FusA‐PM1_fd_ complex.

**Data S3.** The structural coordinates of 2^nd^‐rank HADDOCK‐generated docking model of the FusA‐PM1_fd_ complex.

**Movie S1.** The animation of the rotational motion of the cytotoxic domains in three conformations of PM structure facilitated by the α‐helix linker. The cartoon representation of PM demonstrates its structural elements, depicting the ferredoxin domain in green, the linker in cyan, the cytotoxic domain in red, and the [2Fe‐2S] cluster as a sphere model.

## Data Availability

The structural data that support these findings are openly available in the wwPDB at https://doi.org/10.2210/pdb8jc1/pdb.
