# Supplementary material for: Crystal structure of pectocin M1 reveals diverse conformations and interactions during its initial step via the ferredoxin uptake system
Source: FEBS Open Bio. 2024 Aug 9;14(10):1731–45. doi: 10.1002/2211-5463.13874 (PMC11452297; doi:10.1002/2211-5463.13874)
Supplement: Supplementary file 1 — Fig. S1. Validation of PM1 purification. Fig. S2. Pectocin M1 crystallization and crystal morphology. Fig. S3. Structural analysis of the FusA–AtFd2 complex obtained via HADDOCK docking simulation. Fig. S4. Superimposed models of the catalytic domain of four PM1 molecules in the crystallographic asymmetric unit (residues = 135–267). Table S1. HADDOCK docking statistics. Table S2. Analysis of collisions of amino acid residues in FusA–Pectocin M model complexes. [file FEB4-14-1731-s005.pdf]

Supplementary Table S1. HADDOCK docking statistics [a].

	Cluster 5	Cluster 10	Cluster 1	Cluster 2	Cluster 7	Cluster 6	Cluster 3	Cluster 4	Cluster 8	Cluster 9
**HADDOCK score**	–96.9 ± 7.8	–73.4 ± 4.1	–66.1 ± 2.1	–62.0 ± 1.2	–61.5 ± 7.8	–59.4 ± 8.2	–52.7 ± 3.2	–50.9 ± 2.3	–44.3 ± 8.2	–39.3 ± 12.1
**Cluster size**	8	4	54	38	6	7	16	9	5	4
**RMSD from overall lowest-energy structure (Å)**	0.8 ± 0.5	3.3 ± 0.3	9.1 ± 0.3	8.4 ± 0.2	7.7 ± 0	7.1 ± 0.2	9.2. ± 0.2	6.4 ± 0.8	6.4 ± 0.3	7.2 ± 0.3
**Van der Waals energy (kcal mol^{-1})**	–59.2 ± 6.9	–51.9 ± 6.3	–50.8 ± 1.5	–49.9 ± 2.0	–39.8 ± 7.5	–45.1 ± 3.5	–39.7 ± 2.1	–36.7 ± 1.1	–43.8 ± 4.5	–20.1 ± 5.1
**Electrostatic energy (kcal mol^{-1})**	–301.3 ± 5.8	–222.3 ± 18.6	–126.1 ± 10.2	–124.4 ± 14.3	–172.7 ± 34.6	–121.9 ± 31.0	–87.8 ± 13.6	–143.2 ± 10.6	–38.6 ± 7.0	–156.2 ± 21.6
**Desolvation energy (kcal mol^{-1})**	14.0 ± 1.6	9.5 ± 2.4	0.2 ± 2.0	3.1 ± 0.7	4.4 ± 1.8	4.8 ± 3.4	–3.4 ± 1.6	5.6 ± 1.0	–1.1 ± 0.9	2.1 ± 1.2
**Restraints violation energy (kcal mol^{-1})**	84.5 ± 13.7	134.2 ± 20.9	97.3 ± 27.1	90.9 ± 21.1	84.4 ± 26.9	52.3 ± 26.9	79.6 ± 26.5	87.3 ± 44.5	82.7 ± 27.9	99.7 ± 45.9
**Buried surface area (Å^2)**	2196.9 ± 210.2	1796.6 ± 116.5	1458.8 ± 33.1	1331.7 ± 47.6	1369.0 ± 97.1	1371.8 ± 138.7	1282.6 ± 29.5	1317.0 ± 76.8	1271.3 ± 26.3	1091.6 ± 98.4
**Z-score**	–2.3	–0.8	–0.4	–0.1	–0.1	0.1	0.5	0.6	1.1	1.4

[a] Docking statistics for each cluster of FusA and $PM1_{fd}$ generated by HADDOCK 2.4. PDB coordinates of the two top-score docking models are available in **Supplementary Data 2** for the 1st model and **Supplementary Data 3** for the 2nd model

Supplementary Table S2. Analysis of collisions of amino acid residues in FusA–Pectocin M model complexes [a].

Complex	Number of clashing residues in FusA	List of clashing residues in FusA	Number of clashing residues in pectocin	List of clashing residues in pectocin
FusA-PM1	208	81,82,83,101,103,104,105,106,107,108,109,110, 111,112,148,149,150,151,152,155,156,157,169, 215,216,217,218,219,220,221,222,223,224,225, 226,227,231,232,233,234,235,236,237,238,239, 240,241,242,243,244,245,246,247,248,249,250, 251,252,270,272,273,274,275,276,277,278,279, 280,281,282,283,284,285,286,286,287,288,289, 290,291,292,293,294,295,297,319,320,321,322, 323,324,325,326,327,328,329,330,331,332,333, 334,335,336,368,369,370,371,372,373,374,375, 376,377,378,379,380,393,394,395,396,397,398, 400,401,403,404,405,406,407,408,409,410,458, 459,460,461,462,463,464,465,466,468,481,482, 483,484,490,491,492,493,494,495,496,497,583, 585,586,587,588,595,601,602,603,688,744,746, 748,753,754,755,756,757,758,772,773,805,806, 807,808,809,810,811,812,828,829,830,831,832, 833,834,835,836,837,838,845,847,848,852,853, 854,855,856,857,858,859,860,861,862	172	9,10,11,12,14,16,28,29,30,31,50,51,52,53,54,65,66,67,69,70, 71,84,86,91,92,94,95,96,97,98,99,100,101,102,103,104,105, 106,107,108,109,110,111,112,113,114,115,116,117,118,119, 120,121,123,124,125,126,127,128,129,130,131,132,133,134, 135,136,137,138,139,140,141,142,143,144,145,146,147,148, 149,150,151,152,153,154,155,156,157,158,159,160,161,162, 163,164,165,166,167,172,173,174,175,176,177,178179,180, 181,182,183,184,185,186,187,188,189,190,191,192,193,194, 195,196,197,198,199,200,201,213,215,216,217,218,219,220, 221,222,223,224,225,226,227,228,229,230,231,232,233,234, 235,239,240,242,243,244,245,246,247,248,249,250,251,252, 253,254,255,256,257,258,259,260,261
FusA-PM2 (PDB ID=4n58)	108	149,150,151,155,156,157,234,282,283,327,330, 331,371,372,373,374,375,376,377,378,378,379, 380,390,391,392,393,395,396,397,398,400,401, 403,405,406,407,408,409,410,411,454,455,456, 457,458,459,460,461,462,463,464,465,466,467, 474,475,476,477,478,479,480,481,482,483,484, 485,486,487,488,489,490,491,492,493,494,495, 496,497,498,499,500,582,583,584,585,586,587, 588,589,590,591,592,593,594,595,596,597,598, 599,600,601,603,772,773,845,847,848	92	7,9,10,11,12,14,28,29,30,31,50,51,52,53,54,66,67,69,70,71,8 4,86,91,92,93,94,95,96,97,98,99,100,101,102,103,104,105,1 06,107,108,109,110,116,117,118,122,123,124,125,126,127,1 28,129,130,131,132,133,134,135,136,137,138,139,140,141,1 42,143,144,227,228,229,230,231,232,233,234,241,242,243,2 44,245,246,247,248,249,250,251,252,253,254,255,256
FusA-PM2 (PDB ID=4n59)	120	149,150,151,154,155,157,234,239,281,282,283, 284,285,286,287,288,324,325,326,327,328,329, 330,331,332,333,334,371,372,373,374,375,376, 377,378,379,380,381,382,386,387,388,389,390, 391,392,393,394,395,396,397,398,399,400,401, 402,403,404,405,406,407,408,409,411,455,456, 457,458,459,460,461,462,463,464,465,466,467, 472,475,476,477,478,479,480,481,482,483,484, 485,486,487,488,489,490,491,492,493,494,495, 497,583,584,585,586,587,588,589,590,591,592, 595,596,597,599,601,772,773,845,847,848	98	7,9,10,11,12,14,28,29,30,31,49,50,51,52,53,54,64,66,67,69,7 0,71,81,84,86,91,92,93,94,95,96,97,98,99,100,101,102,103,1 04,105,106,107,108,109,110,111,112,113,114,116,118,124,1 25,126,127,128,129,130,131,132,133,134,135,136,137,138,1 51,184,185,186,187,188,189,190,191,192,193,194,195,196,1 97,198,227,228,229,230,231,232,233,246,247,248,249,250,2 51,252,253,254

[a] Analysis of collisions among amino acid residues in complexes of FusA and different conformations of PM obtained from docking full-length crystal structures of PM into the ferredoxin domain of PM1 in the model of the FusA–PM1$_{fd}$ complex. The number and list of clashing residues in FusA–PM1, FusA–PM2 (PDB ID=4n58), and FusA–PM2 (PDB ID=4n59) are listed. The analysis was performed using the Contact program in the CCP4 software suite.

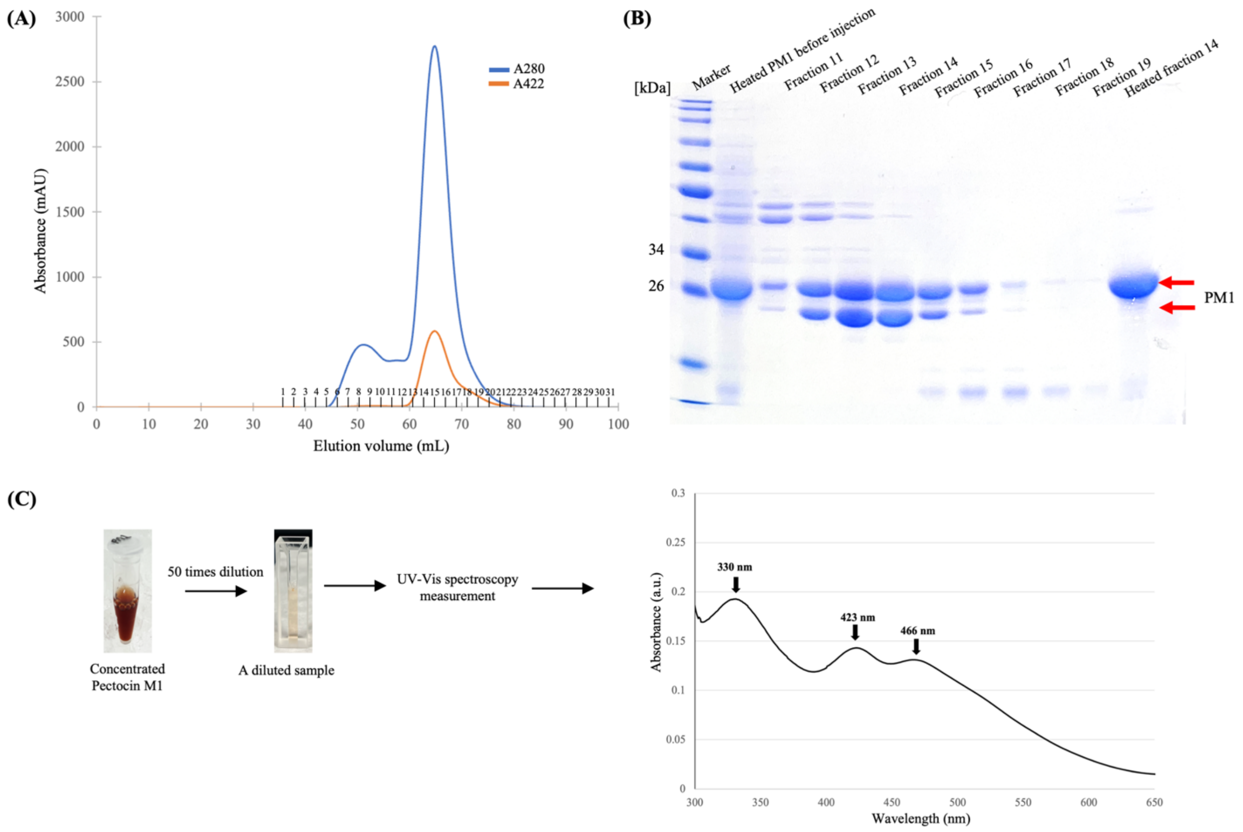

Supplementary Figure S1. Validation of PM1 purification. (A) Superdex 75 10/60 gel filtration profile of purified PM1 monitored at wavelengths of 280 and 422 nm. (B) SDS-PAGE analysis of PM1 samples before and after gel filtration, and with and without heat treatment. Red arrows indicate two bands corresponding to PM1. (C) Workflow detailing UV-Vis spectroscopy measurement of a 50-fold diluted PM1 solution after the final purification step.

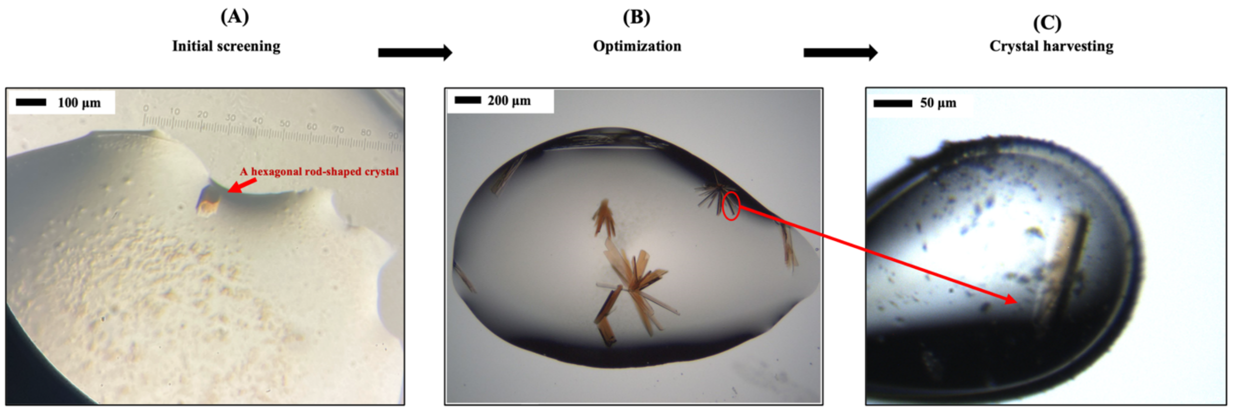

Supplementary Figure S2. Pectocin M1 crystallization and crystal morphology. (A) Single crystal (~80 x 20 µm) of PM1 obtained after 2 days during the initial screening. (B) Brown crystals with rod and plate clusters formed after 21 days of optimization using 0.1 M HEPES at pH 7.5 and 19.2% w/v poly (acrylic acid sodium salt) 5100. (C) The indicated rod-shaped crystal (~150 x 40 µm) in (B) was harvested using 20% glycerol as a cryoprotectant and used for subsequent data collection.

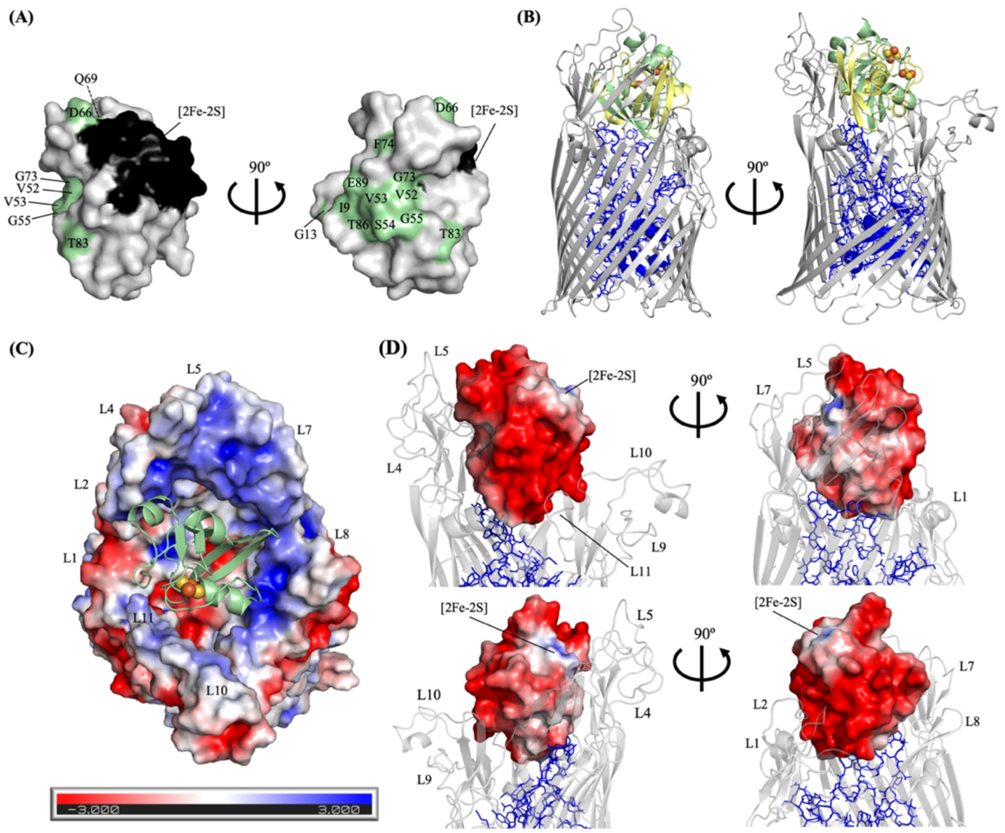

Supplementary Figure S3. Structural analysis of the FusA–AtFd2 complex obtained via HADDOCK docking simulation. (A) Active residues assigned in the simulation (indicated on a gray background in Fig. 1A) are mapped onto the surface of the 3D structure of AtFd2 (light green). (B) Crystal structure of FusA (gray) with the top two models of AtFd2 superimposed (pale green, 1st rank; pale yellow, 2nd rank). The plug domain of FusA is shown as blue sticks and cartoons; the [2Fe-2S] cluster is shown as spheres. (C) Top scored model of the FusA–AtFd2 complex; FusA is shown as a molecular surface mapped with electrostatic potential; the crystal structure of AtFd2 is shown as a green cartoon model. (D) AtFd2 molecular surface mapped with electrostatic potential docked to FusA viewing from the front (top panel) and the back (bottom panel) with the right panel rotated in 90 ° each, shown as a white cartoon model with its plug domain shown as a blue stick and cartoon model.

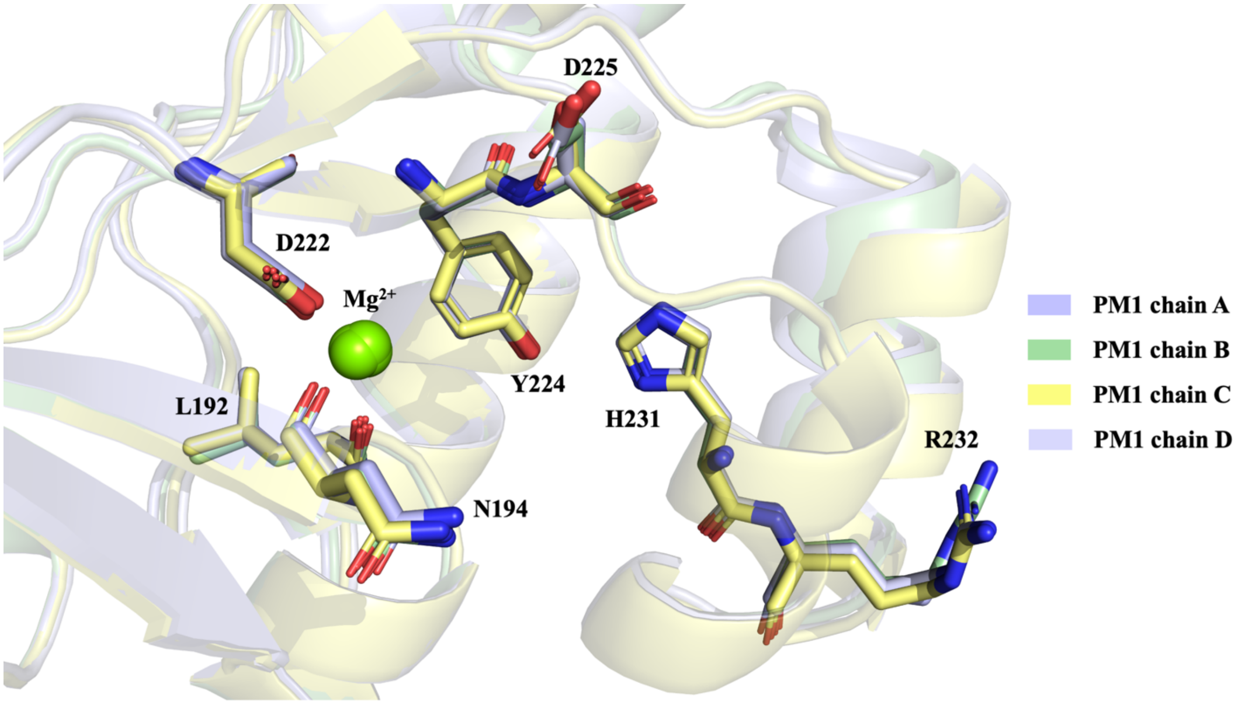

Supplementary Figure S4. Superimposed models of the catalytic domain of four PM1 molecules in the crystallographic asymmetric unit (residues = 135–267). The key amino acids in the active site of the catalytic domain PM1 are represented by sticks.
